# Upregulation of MUC5AC production and deposition of LEWIS determinants by *HELICOBACTER PYLORI* facilitate gastric tissue colonization and the maintenance of infection

**DOI:** 10.1186/s12929-019-0515-z

**Published:** 2019-03-06

**Authors:** Weronika Gonciarz, Maria Walencka, Anthony P. Moran, Krzysztof Hinc, Michał Obuchowski, Magdalena Chmiela

**Affiliations:** 1 Division of Gastroimmunology, Department of Immunology and Infectious Biology, Institute of Microbiology, Biotechnology and Immunology, Faculty of Biology and Environmental Protection, University of Łódź, Banacha 12/16, 90-237 Łódź, Poland; 2 Department of Microbiology, School of Natural Sciences, National University of Ireland Galway, Galway, Ireland; 3 Laboratory of Molecular Bacteriology, Intercollegiate Faculty of Biotechnology UG-MUG, Medical University of Gdańsk, 80-210 Gdańsk, Poland

**Keywords:** MUC5AC, LewisX/Y, *H. pylori*, Guinea pigs

## Abstract

**Background:**

*Helicobacter pylori* bacteria colonize human gastric mucosa, cause chronic inflammation, peptic ulcers and gastric cancer. Colonization is mediated by *H. pylori* adhesins, which preferentially bind mucin 5 (MUC5AC) and Lewis (Le) determinants. The aim of this study was to evaluate the influence of *H. pylori* and their components on MUC5AC production and deposition of LeX/LeY in gastric epithelial cells in relation to bacterial adhesion using *Caviae porcellus* primary gastric epithelial cells and an in vivo model of experimental *H. pylori* infection in these animals.

**Methods:**

MUCA5C and LeX/LeY were induced in vitro by live *H. pylori* reference strain CCUG 17874 (2 × 10^7^ CFU/ml), *H. pylori* glycine acid extract (GE), 10 μg/ml; cytotoxin associated gene A (CagA) protein, 1 μl/ml; UreA urease subunit, 5 μg/ml; lipopolysaccharide (LPS) 25 ng/ml and imaged by fluorescence microscopy after anti-MUC5AC or anti-LeX/LeY FITC antibody staining. Bacterial adhesion was imaged by using anti-*H. pylori* FITC antibodies. The animals were inoculated per os with *H. pylori* (3 times in 2 days intervals, 1 × 10^10^ CFU/ml). After 7 or 28 days an infection and inflammation were assessed by histological, serological and molecular methods. Gastric tissue sections of infected and control animals were screend for MUCA5C and LeX, and *H. pylori* adhesion as above.

**Results:**

MUC5AC production and deposition of Lewis determinants, especially LeX were upregulated in the milieu of live *H. pylori* as well as GE, CagA, UreA or LPS in vitro and in vivo during infection, more effectively in the acute (7 days) than in the chronic (28 days) phase of infection. This was related to enhanced adhesion of *H. pylori*, which was abrogated by anti-MUC5AC and anti-LeX or anti-LeY antibody treatment.

**Conclusions:**

Modulation of MUCA5C production and LeX/LeY deposition in the gastric mucosa by *H. pylori* can significantly increase gastric tissue colonization during *H. pylori* infection.

## Introduction

The gastric mucosa is a physical barrier covered with a mucus layer, which protects stomach against harmful chemical, enzymatic, microbiological and mechanical factors [1, 2]. The integrity of gastric epithelial cells is maintained by tight junctions whereas cell proliferation promotes a renewal of this layer. Also the outer leyer of gastric mucosa loosely adheres to epithelium and is constantly removed and renewed***,*** which protects a movement of pathogens to the stomach basement membrane [3, 4]. However, *Helicobacter pylori* Gram-negative microaerophilic rods, discovered by Warren and Marshall in 1983, are able to penetrate through gastric mucosa due to their spiral shape and flagella [5–8]. *H. pylori* can change the structure of mucus by thioredoxin, which reduces the disulfide bonds of mucins, thereby diminishing the capacity of mucin gel-formation [9]. Colonization of gastric mucosa is facilitated by urease, which generates ammonia neutralizing the acidic environment of the stomach [10, 11], and is followed by the reduction of mucus viscosity and elasticity [12, 13]. In the case of strains positive for cytotoxin associated gene A (CagA) protein, colonization results in disruption the cell junctions and loss of cell polarity [14–16]. The cell damage induces infiltration of inflammatory cells including neutrophils, macrophages and lymphocytes and excessive gastrin versus decreased somatostatin production [17, 18]. This results in an increased secretion of hydrochloric acid and movement of the gastric content with microorganisms into the duodenum, thus increasing the colonization area [19]. *H. pylori* infection may be asymptomatic or symptomatic with lesions, which arise after prolonged exposure to hydrochloric acid in the case of gastric or duodenal ulcers [20–22]. Initially active gastritis induced by *H. pylori* can transform into atrophic gastritis and then to neoplastic lesions and promote the development of mucosa-associated lymphoid tissue (MALT) lymphoma (0.1%), or gastric adenocarcinoma [23–25]. Long-term colonization of gastric epithelial cells by *H. pylori* depends on various *H. pylori* surface adhesins including: the blood group antigen-binding adhesin A (BabA) and sialic acid binding adhesin (SabA), adherence-associated lipoprotein A (AlpA) and B (AlpB), *Helicobacter* outer membrane protein Z (HopZ), outer membrane protein A (OpiA) and proteins binding the host extracellular matrix components (ECM) [26–32]. Lipopolysaccharide (LPS) of *H. pylori* contains long-chain fatty acids and in the O-specific part there are sugar moieties similar to human Lewis (Le) blood-group antigens, which interact with corresponding sugar compounds of gastric mucin [33–37]. Among mucins, which are exposed on gastric epithelial cells the secretory mucin 5 (MUC5AC) is dominanting. This mucin can undergo modification to Le antigens, which play a role of receptors to *H. pylori* during infection [2, 37]. In humans Le antigens, especially Leb and LeX as well as sialylated LeX are the major putative receptors on gastric epithelial cells of the *H. pylori* infected host that bind *H. pylori* via BabA and SabA, respectively [27, 38–40]. A heterogeneity among *H. pylori* strains in expression of the outer membrane protein BabA is postulated as pathogen fitness to diverse human population [41]. Bäckström et al. (2004) showed that 70% of Swedish and U.S. *H. pylori* clinical isolates exhibited Leb binding but the *babA* gene was present in each of 10 Leb non-binding strains. Leb non-binding strains also possess silent *babA* gene, which can be activated by recombination to *babB* locus. At this locus, a BabB/A chimeric adhesin is expressed and is subject to phase varaition (ON/OFF switching) [42]. Concerning SabA it’s expression is also controlled via phase variation and the ArsRS signal transduction signal [43]. Various studies showed that MUC5AC with deposition of Le determinants is a key component of human gastric mucosa involved in *H. pylori* colonization. However, the knowledge about the influence of live bacteria or their soluble components on MUC5AC production as well as Le antigens deposition and management of *H. pylori* attachment to gastric epithelial cells and then colonization is insufficient. In this study, by using two *Caviae porcellus* (guinea pig) models: a model of primary gastric epithelial cells and a model of experimental *H. pylori* infection, we focused on the relation between MUC5AC and LeX/LeY production in response to gastric epithelial cell exposure in vivo or in vitro to *H. pylori* reference LeX/Y positive strain or soluble components of these bacteria, and the effectiveness of epithelial cell colonization. We considerd both the host and bacterial LeX/LeY components in the course of *H. pylori* adhesion to gastric epithelial cells on guinea pig model.

## Materials and methods

### *H. pylori* strains and culture conditions

*Helicobacter pylori* reference strain CCUG 17874 (Culture Collection, University of Gothenburg, Gothenburg, Sweden) positive for vacuolating cytotoxin A (VacA) and CagA proteins as well as for LeX and LeY determinants in LPS, which were confirmed previously by immunotyping with anti-LeX or anti-LeY antibodies [44], was used in this study. *H. pylori* bacteria were stored at − 80 °C in Trypticase Soy Broth (TBS) containing 10% glycerol. Bacteria were cultured under microaerophilic conditions according to the previously described procedure [45].

### Stimuli

Glycine acid extract (GE) from the reference *H. pylori* strain CCUG 17874 was a source of surface *H. pylori* antigens, which were extracted using 0.2 mol/l glycine buffer, pH 2.2, as previously described [46, 47]. Protein composition of GE was evaluated by SDS-PAGE electrophoresis and Western blot - Immuno blot (Milenia® Blot *H. pylori*, DPC Biermann, GmbH, Bad Nauheim, Germany), with the reference serum samples from patients infected with *H. pylori* [48]. Major proteins in GE recognized by sera from *H. pylori* infected patients were: 120 kDa (CagA), 87 kDa (VacA), 66 kDa (UreB), 60 kDa (Hsp), 29 kDa (UreA), between 66 and 22 kDa. The protein concentration in GE was 600 μg/ml (NanoDrop 2000c Spectrophotometer, ThermoScientific, Wlatman, WY, United States). GE contained < 0.001 EU/ml of LPS, as shown by the chromogenic *Limulus amebocyte* lysate test (Lonza, Braine-Alleud, Belgium). GE was applied at 10 μg/ml. Recombinant CagA protein - rCagA (a kind gift from Antonello Covacci, IRIS, Siena, Italy), was used at 1 μg/ml. A recombinant fragment of CagA antigen of *H. pylori*: nt 2777 to nt 3465 of *cagA* gene was used. It was expressed (QIAexpress System, Qiagen, Hilden, Germany) in *E. coli* as a fusion protein (about 26 kDa size) with a 6 His-tail in front of a 230 aa polypeptide of CagA. The protein was purified by the Ni^2 + −^NTA agarose column, and checked for serological activity by the enzyme immunoassay [49, 50]. Due to patent claims of *H. pylori* urease the UreA subunit from *H. acinonychis* isolated from the acidic environment of cheetah stomach was used as a homoloque of *H. pylori* protein (97% homology). The urease gene was amplified by PCR, as previously described [51], using chromosomal DNA as a template and oligonucleotides hisure-A-up and hisure-A-dn as primers. DNA encoding six histidines (His6-tag) was carried by oligonucleotide hisure-A-dn. The obtained PCR product of 737 bp was digested with enzymes *KpnI* and *NheI* and cloned into the commercial vector pBAD (Stratagene, California, United States). The resulting plasmid, pMD1, was verified by restriction analysis and nucleotide sequencing. pMD1 was used to transform the *E. coli* strain DH5α and the recombinant strain was used to overproduce UreA by the addition of arabinose 0.05%. A 27 kDa protein was visualized on a coomassie blue stained gel and purified on Ni-NTA superflow agarose (Qiagen) followed by gel filtration on Superose 6resin. UreA was used at 5 μg/ml. LPS from the reference strain of *H. pylori* CCUG 17874 was prepared by hot phenol-water extraction. Whole cell lysates were pretreated with proteinase K (Sigma, St Louis, MI, United States). Crude extraction of LPS from bacteria was performed with 45% aqueous phenol at 68 °C for 30 min. The LPS preparation was purified by the treatment with RNase A, DNase II and proteinase K (Sigma), and by ultracentrifugation at 100000×*g* at 4 °C, for 18 h [52–54]. *H. pylori* LPS and control LPS of *E. coli* (serotype O55: B5; Sigma) were used at 25 ng/ml. The antigen concentrations were adjusted experimentally or adopted from previous studies [55–57].

### *H. pylori* infection in Guinea pigs

Adult, three-month-old, 400–600 g of weight male Himalayan *Cavia porcellus* (guinea pigs) were used in the experiments. Animals were bred in the Animal House at the Faculty of Biology and Environmental Protection, University of Lodz (Poland), kept in cages with free access to drinking water and fed with standard chow. The experiments were approved by the Local Ethics Committee LKE9 (Decision 58/ŁB45/2016). The animal study groups consisted of guinea pigs (*n* = 15), which were inoculated per os three times (at two-day intervals) with 1 ml of sterile complete *Brucella* broth using a feeding needle (control group; *n* = 5), or with 1 ml of freshly prepared suspension of *H. pylori* (1 × 10^10^ colony forming units - CFU)/ml; *n* = 10). Before the administration of complete *Brucella* broth or *H. pylori* suspension, the animals obtained orally 1 ml of 0.2 M NaHCO_3_ to quickly neutralize the acidic pH of the stomach. 7 and 28 days after the last dose of *H. pylori* the guinea pigs were euthanized, and biological samples were collected for further study. *H. pylori* status was confirmed according to previously described methods [45, 57]. Anti-*H. pylori* IgG/IgM antibody content in the serum samples, and the level of *H. pylori* antigens in the stool were detected by enzyme-linked immunosorbent assay (ELISA). Histopathological methods were used to detect *Helicobacter*-like organisms (HLO) and inflammation whereas polymerase chain reaction (PCR) was applied to detected *cagA* and *ureC* gene sequences in the guinea pigs gastric tissue.

### Cell cultures

Primary gastric epithelial cells were obtained according to the previously described procedures [58–60], with some modifications. The guinea pig was euthanized by overdosing sodium barbiturate (Morbital, Biowet, Poland), the stomach was isolated, rinsed with Hank’s Balanced Salt Solution (HBSS), pH 7.4 (Sigma) supplemented with penicillin (100 U/ml), streptomycin (100 μg/ml) and amphotericin B (0.025 mg/ml) (Polfa Tarchomin S.A., Warszawa, Poland), homogenized and then tripsinized (15 min., room temp). Next, 2% bovine serum albumin (BSA) solution (Sigma) in HBSS was added to homogenates, which were then centrifuged at 3000×*g* for 15 min. The supernatant was removed and the pellet was suspended in 5% BSA in Dulbecco’s Modified Eagle’s *medium (*DMEM) (Sigma) supplemented with penicillin (100 U/ml), streptomycin (100 μg/ml) and amphotericin B (0.025 mg/ml) (Polfa). Cell suspensions 2 × 10^6^ cells/ml were added to the wells of 6-well plates (Becton Dickinson, USA), and incubated for 24 h (5% CO_2_, 37 °C) to adhere. Unbound cells were washed out with phosphate-buffered saline (PBS), pH 7.4 and the remaining cells were cultured with DMEM and Ham’s F-12 1: 1 (Sigma) supplemented with 10% fetal calf serum (FCS), 1% N-2-hydroxyethylpiperazine-N-2-ethane sulfonicacid (HEPES) (Sigma), penicillin (100 U/ml), streptomycin (100 μg/ml), amphotericin B (0.025 μg/ml), L-glutamine (2 mM/ml) (Polfa), epidermal growth factor (Sigma) 0.01 μg/ml and 0.005% dexamethasone solution in complete RPMI-1640 culture medium (cRPMI) (Sigma). Every 48 h, the medium was changed and after 8 days of cultivation, confluent monolayers were treated with 0.25% trypsin (BIOMED-LUBLIN, Lublin, Poland) and transferred to breeding bottles. After 14 days cells were used for testing.

### Evaluation of MUC5AC and LeX or LeY dependent *H. pylori* adhesion to Guinea pig primary gastric epithelial cells

Primary gastric epithelial cells (1 × 10^6^ cell/ml, 1 ml) were cultured in DMEM: F12 medium (37 °C, 5% CO_2_) in wells of a 6-well culture plates containing glass coverslips (ThermoScientific, USA). Unstimulated cells (control) or cells stimulated for 24 h with selected *H. pylori* antigens: GE 10 μg /ml, CagA 1 μg/ml, UreA 5 μg/ml and *H. pylori/E. coli* LPS (control), 25 ng/ml or for 2 h with live *H. pylori* (2 × 10^7^ CFU/ml) were fixed with 4% formaldehyde solution, 20 min, room temp., and then washed 3 times in PBS. For increasing cell permeability cells were treated with 0.2% Triton-X-100 for 10 min and then washed 3 times as before. After blocking an unbound glass with 3% BSA in PBS, 1 h, cells were used for further procedures.The production of mucin and Le X or Le Y determinants was evaluated by incubating cells with primary mouse anti-MUC5AC antibodies (MyBiosource, USA), diluted 1: 200 in PBS/BSA or anti-Le X/Le Y antibodies (Dako, Glostrup, Denmark), 2 μg/ml, for 2 h at room temp. After washing the excess of antibodies away, cells were incubated for 1 h in the dark with sheep anti-mouse immunoglobulins antibodies (100 μl) conjugated with fluorescein isothiocyanate (FITC) (Sigma), diluted 1: 64 in PBS/BSA. In parallel, cells exposed to *H. pylori* antigens, treated or not treated with anti-MUC5A antibodies or anti- LeX/LeY antibodies (Dako), and then incubated with live *H. pylori* (1 ml, 2 × 10^7^ CFU/ml, 2 h, 37 °C, 5% CO_2_) were used. The unbound bacteria were washed out, and cells were incubated for 1 h with rabbit anti-*H. pylori* antibodies - FITC (100 μl, 1:200 in BSA/PBS) (MyBiosource). Cells were then stained with Texas Red-X phalloidin (Thermo Scientific) solution (2.5 μg/ml), 15 min at room temp, and with 4′,6-diamidino-2-phenylindole (DAPI; Sigma) solution (2.5 μg/ml) at the same conditions. Slides were mounted in a DPX balsam (Surgipath, Great Britan) and then imaged in a confocal microscope (Leica TCS SPE) at a wavelength for FITC 495 nm (excitation), 519 nm (emission); for phalloidyne 591 nm (excitation), 608 nm (emission) and for DAPI 358 nm (excitation), 461 nm (emission), at 640× magnification. In all experiments, controls of antibodies were set to exclude non-specific reactions. Three independent experiments and replications were performed.The production of MUC5A or LeX/LeY was assessed quantitatively on the basis of green fluorescence intensity measured using a multifunctional Victor 2 reader (Wallac, Oy, Turku, Finland), and by fluorescence imaging [56, 61].

### Detection of MUC5AC, LeX and *H. pylori* bound to the Guinea pig gastric tissue

Guinea pig gastric tissue specimens (7 and 28 days after inoculation with *H. pylori* or control) fixed in formalin, embedded in paraffin were cuted to tissue sections (4 μm), which were placed on the adhesive slide and then deparaffinized. For antigen exposure they were heated (95–100 °C) in sodium citrate buffer pH 6.0, 20 min, cooled to room temp. and washed 3 times in PBS. The slides were blocked for 1 h (BSA/PBS) and washed as above. MUC5AC was stained with mouse anti-MUC5AC antibodies 1:100 (MyBiosource, USA), whereas LeX with anti-LeX antibodies (DAKO), 2 μg/ml, overnight at 4 °C. After washing 5 times in Tris-Buffered Saline with Tween 20 (TBST), pH 7.4, sheep anti-mouse IgG antibodies conjugated with FITC (Sigma),1:64, were added on slides, which were incubated for 20 min at room temp. Cell nuclei were stained with DAPI solution (2.5 μg/ml) or phalloidine, 15 min, at room temp, as above. Intensity of fluorescence was measured by using the software ImageJ 1.48v (National Institute of Health, United States) under fluorescence microscope (Zeiss, Axio Scope, A1, Germany) at an appropriate wavelength: for FITC 495 nm (excitation)/519 nm (emission); for phalloidyne 591 nm (excitation)/608 nm (emission), and for DAPI 358 nm (excitation)/461 nm (emission), at 100× magnification [56, 61]. The ability of *H. pylori* to bind MUC5AC or LeX was assessed on tissue sections preincubated with blocking anti-MUC5AC or anti-LeX antibodies, or control unblocked sections, which were then incubated with *H. pylori* (2 × 10^7^ CFU/ml) for 2 h. After washing the specimens were stained with rabbit anti-*H. pylori* antibodies FITC and with DAPI, and fluorescence intensity was evaluated by imaging in fluorescence microscope as above.

### Statistical analysis

Data were expressed as the mean ± standard deviation (SD). The differences between groups were tested using the non-parametric Mann-Whitney *U* test or the Kruskal-Wallis test. For statistical analysis the Statistica 12 PL software was used. Results were considered statistically significant when *p* < 0.05.

## Results

### MUC5AC and LeX/LeY production by Guinea pigs gastric epithelial cells in the milieu of live *H. pylori* or soluble compounds of these bacteria in relation to *H. pylori* adhesion – In vitro and in vivo models

**Fig. 1:**
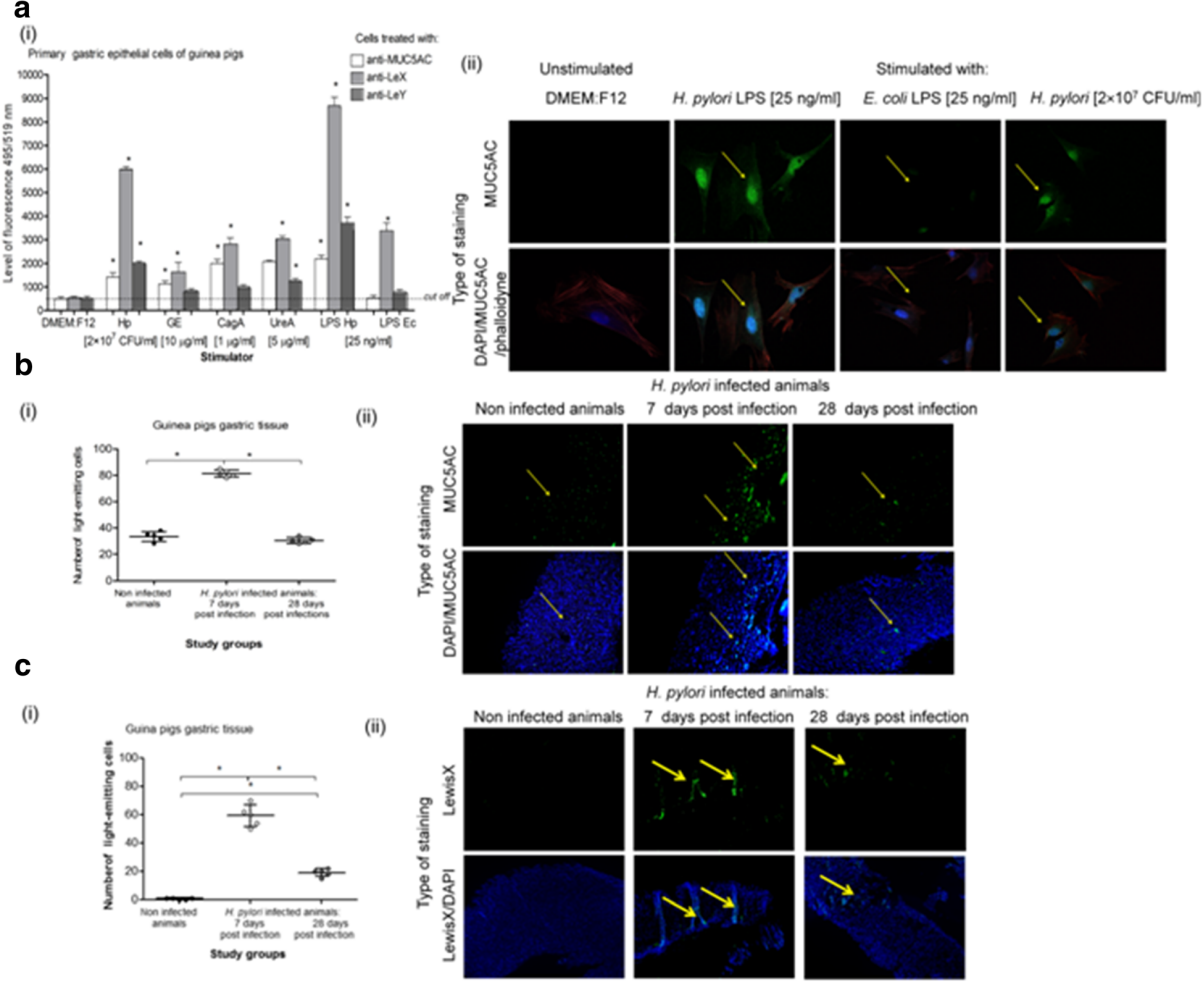
MUC5AC and Lewis X/Y production by guinea pig gastric epithelial cells. MUC5AC and Lewix X/Y was evaluated on the basis of the intensity of fluorescence of guinea pig primary gastric epithelial cells or gastric tissue specimens stained with mouse anti-MUC5AC or anti-LeX/anti-LeY antibodies (Ab) and secondary antibodies conjugated with fluoresceine isothiocyanate (FITC) and counterstained with DAPI or phalloidine. a- guinea pig primary gastric epithelial cells non treated (in culture medium alone) or treated with selected *H. pylori* components: for 24 h with glycine acid extract – GE (10 μg/ml), urease subunite A – UreA (5 μg/ml), cytotoxin associated gene A (CagA) protein (1 μg/ml), *H. pylori* lipopolysaccharide (LPS Hp) or *E.coli* LPS –LPS Ec (25 ng/ml) or for 2 h with live *H. pylori* - Hp(2 × 10^7^ colony forming units - CFU/ml), (i) intensity of fluorescence measured in a fluorescence reader at 495 nm (excitation) and 519 nm (emission), mean values ± SD. * Statistical significance for *p* < 0.05 assessed by non parametric *U* Mann-Whitney test, (ii) representative images from a confocal microscope (Leica TCS SPE) at wavelengths: FITC - 495 nm excitation and 519 nm emission, DAPI – 345 nm excitation and 455 nm emission, phalloidine – 591 nm excitation and 608 nm emission (630 × magnification). b MUC5AC and c Lewis X imaging in the gastric tissue of guinea pigs experimentally infected with *H. pylori* (7 and 28 days after inoculation, *n* = 10) or control animals (*n* = 5), (bi, ci) intensity of fluorescence measured using the software ImageJ version 1.48v (National Institute of Health, United States) at 495 na (excitation) and 519 nm (emission), mean values±SD. Statistical significance for *p* < 0.05 assessed by the non parametric Kruskal-Wallis test. * Statistically siginificant values for infected animals (7 and 28 days after inoculation) vs control animals and for infected animals 7 days post infection vs 28 days after the last inoculation with *H. pylori*, b ii representattive images of MUC5AC production and c ii representative images of LeX production in guinea pigs gastric tissue from fluorescence microscope (Axio Scope A1, Zeiss, Germany) at wavelengths: FITC 495 nm excitation and 519 nm emission, DAPI 345 nm excitation and 455 nm emission, (100 × magnification)

We were focused on answering the question whether live *H. pylori* rods or their soluble antigens modulate MUC5AC production by the gastric epithelial cells and LeX/LeY deposition, and how it influences the process of colonization of gastric mucosa by *H. pylori* using an in vitro model of primary gastric epithelial cells derived from the guinea pig gastric tissue. We also used an in vivo model of experimental infection with *H. pylori* in these animals. Production of MUC5AC was significantly increased after 24 h stimulation of guinea pig primary gastric epithelial cells with *H. pylori* surface components GE (10 μg/ml), CagA (1 μg/ml), UreA (5 μg/ml), *H. pylori* LPS (25 ng/ml) as well as after 2 h stimulation with live *H. pylori* (2 × 10^7^ CFU/ml) (Fig. 1a), *p* < 0.05 in Kruskal-Wallis test. The highest fluorescence after staining the cells with anti-MUC5AC and secondary FITC labeled antibodies was demonstrated for cells treated with *H. pylori* LPS. Increased production of MUC5AC was corelated with the elevated levels of LeX in all variants of cell cultures, p < 0.05 in Kruskal-Wallis test, especially in the cell cultures treated with live *H. pylori* or *H. pylori* LPS. Deposition of LeY in primary gastric epithelial cells was elevated in cell cultures exposed to whole *H. pylori*, UreA and *H. pylori* LPS (Fig. 1a i). Similarly, MUC5AC production increased in the gastric tissue of guinea pigs inoculated experimentaly with *H. pylori* (Fig. 1b). Only in the gastric mucosa of infected but not of uninfected animals the gastric tissue was positive for HLO, *ureC/cagA* and infiltrated by immunocompetent cells. After 28 days from inoculation of animals with *H. pylori* the number of eosinophils and lymphocytes increased, which indicated the development of chronic inflammatory response. *H. pylori* antigens were detected in stool samples and anti-*H. pylori* antibodies in the sera of infected but not of uninfected animals (7 and 28 days post infection). Significantly higher levels of MUC5AC were demonstrated in animals 7 (acute phase of infection) than 28 days after the last *H. pylori* inoculation (chronic phase of infection) (Fig. 1b i, 1b ii). The production of MUC5AC was linked with the elevated deposition of LeX in gastric tissue, which was stronger after 7 than 28 days post infection (Fig. 1 c i, ii), *p* < 0.05 in Kruskal-Wallis test.

**Fig. 2:**
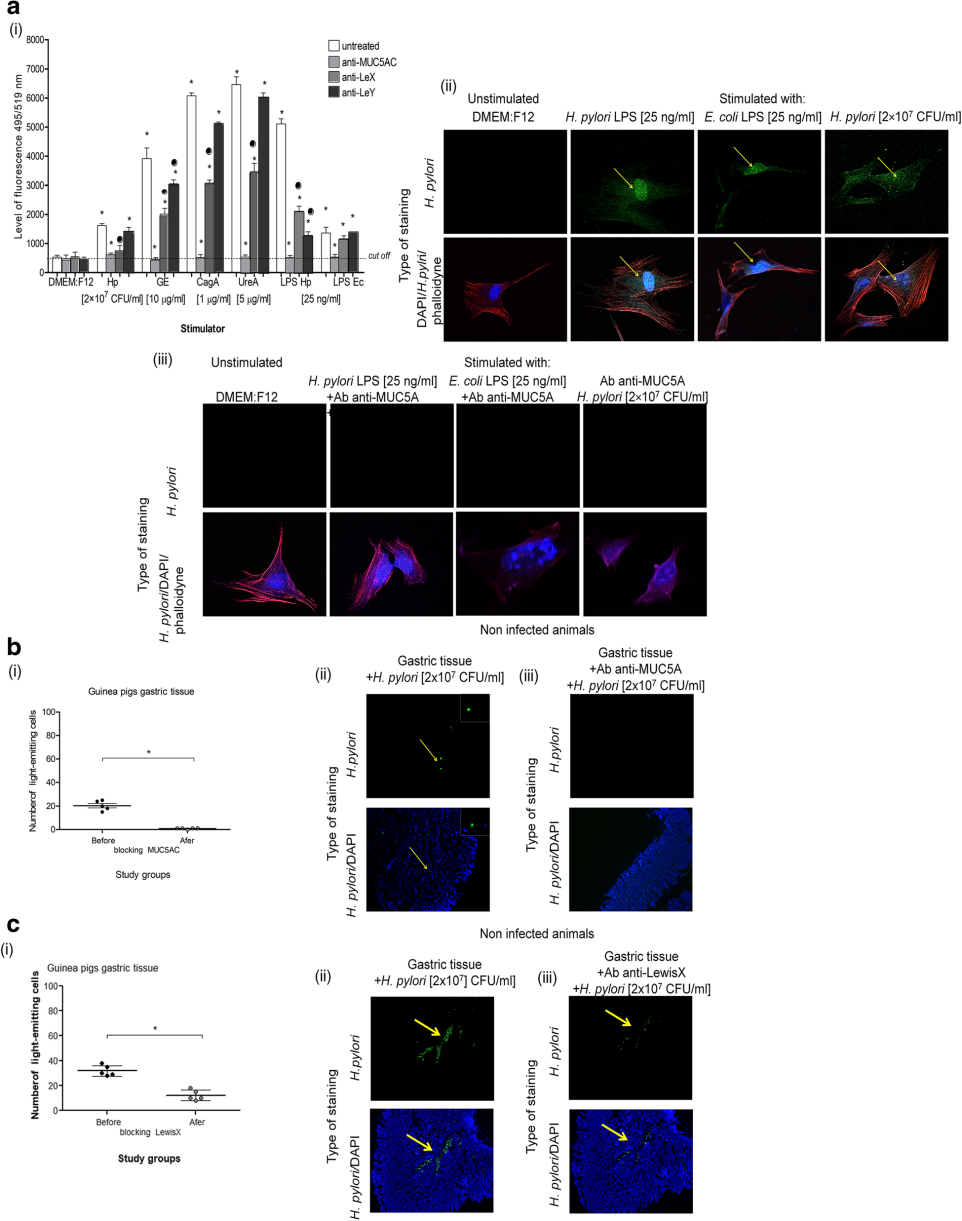
Adhesion of *H. pylori* to guinea pig gastric epithelial cells mediated by MUC5AC mucin and Lewis (Le)X/LeY determinants. Binding of *H. pylori* to guinea pig primary gastric epithelial cells and gastric tissue specimens was evaluated by imaging *H. pylori* stained with anti-*H. pylori* antibodies (Ab) conjugated with fluorescein isothiocyanate (FITC) in a fluorescence reader or confocal microscope, counterstained with DAPI or phalloidine. a – guinea pig primary gastric epithelial cells non treated (in culture medium alone) or treated with selected *H. pylori* components: for 24 h with glycine acid extract – GE (10 μg/ml), urease subunite A – UreA (5 μg/ml), cytotoxin associated gene A (CagA) protein (1 μg/ml), *H. pylori* lipopolysaccharide (LPS Hp) or *E.coli* LPS –LPS Ec (25 ng/ml) or for 2 h with live *H. pylori* – Hp (2 × 10^7^ colony forming units - CFU/ml) were prepared. Further, cells non-treated (control of adhesion) or blocked with anti-MUC5AC or with anti-LeX/LeY antibodies were used in adhesion assay. a i – the intensity of fluorescence measured in a fluorescence reader at 495 nm (excitation) and 519 nm (emission), mean values ±SD. * Statistical significance for cells treated with an individual component *vs *untreated cells or ^#^ treated with individual antibody vs untreated cells (control cells), p < 0.05 in non parametric *U* Mann-Whitney test. Representative images of primary gastric epithelial cells untreated (a ii) or treated (a iii) with anti-MUC5AC antibodies before *H. pylori* binding experiments, stained with anti-*H. pylori* FITC antibodies in confocal microscope (Leica TCS SPE), at 495 nm excitation and 519 emission for FITC, 345 excitation/455 emission for DAPI and 591 nm excitation/608 nm emission for phalloidine (640 magnification). Gastric tissue specimens from non infected guinea pigs untreated or treated with anti-MUC5AC (b) or anti-LeX (c) blocking antibodies before 2 h exposure to live *H. pylori* (2 × 10^7^ colony forming units (CFU)/ml). bi and Ci – intensity of fluorescence measured using the software ImageJ version 1.48v (National Institute of Health, United States) at 495 excitation/519 emission, mean values ±SD. *Statistically significant values for gastric tissue blocked with anti-MUC5AC or anti-LeX antibodies vs unblocked specimens. Representative images of *H. pylori* adhesion to the guinea pig gastric tissue non treated with anti-MUC5AC (bii) or anti-LeX (cii) antibodies vs gastric tissue treated with such antibodies (b iii, iii) from fluorescence microscope (Axio Scope A1, Zeiss, Germany), at 495 nm excitation/519 nm emission for FITC, 345 nm excitation/455 nm emission for DAPI and 591 nm excitation/608 nm emission for phalloidine (100 × magnification)

The mucin production and LeX or LeY deposition were then compared to the effectiveness of *H. pylori* adhesion to guinea pig gastric epithelial cells in vitro and in vivo. To determine whether MUC5AC or LeX/LeY mediated the adhesion of *H. pylori* to the guinea pig gastric epithelium we used guinea pig primary gastric epithelial cells preincubated or not with *H. pylori* components and then untreated or treated with anti-MUC5AC, anti-LeX or anti-LeY blocking antibodies (Fig. 2a i, ii, iii) before the exposure to live *H. pylori*. The results presented in Fig. 2a i, ii, iii show that increased adhesion of *H. pylori* to guinea pig primary gastric epithelial cells was related to the elevated production of MUC5AC in these cells, in response to live *H. pylori* and soluble components of these bacteria, and was completely abrogated by anti-MUC5AC antibodies (Fig. 2a i, ii, iii). Similarly, Fig. 2b i, ii show the results for guinea pig gastric tissue specimens untreated or treated with anti-MUC5AC antibodies and then exposed to live *H. pylori* in vitro*.* The attachment of *H. pylori* to guinea pig gastric tissue specimens was diminished by the pretreatment of tissue sections with anti-MUC5AC antibodies (Fig. 2b i, ii, iii). Furthermore, it was showed that LeX and LeY determinants mediated the binding of *H. pylori* to primary gastric epithelial cells because *H. pylori* binding to these cells was significantly diminished by pretreatment the cells with anti-LeX or anti-LeY antibodies (Fig. 2a i). Similarly, the attachment of *H. pylori* to gastric tissue specimens was abrogated after treatment of specimens with anti-LeX antibodies (Fig. 2c i, ii, iii).

Live *H. pylori* stimulated MUC5AC significantly higher that of *E.coli* LPS. Similarly, the deposition of LeX determinants was higher in response to live *H. pylori* than to LPS *E.coli* (Fig. 1a i). The adhesion assay showed that MUC5AC, which was exposed on the cells treated with live *H. pylori* or LPS *E.coli* was used by *H. pylori* for binding. This was confirmed by blocking of *H. pylori* attachment with anti-MUC5AC (Fig. 2 b i). However, it is possible that *E. coli* LPS could stimulated other than LeX or LeY determinats, not considered in this study, that could mediate adhesion of *H. pylori*.

**Fig. 3:**
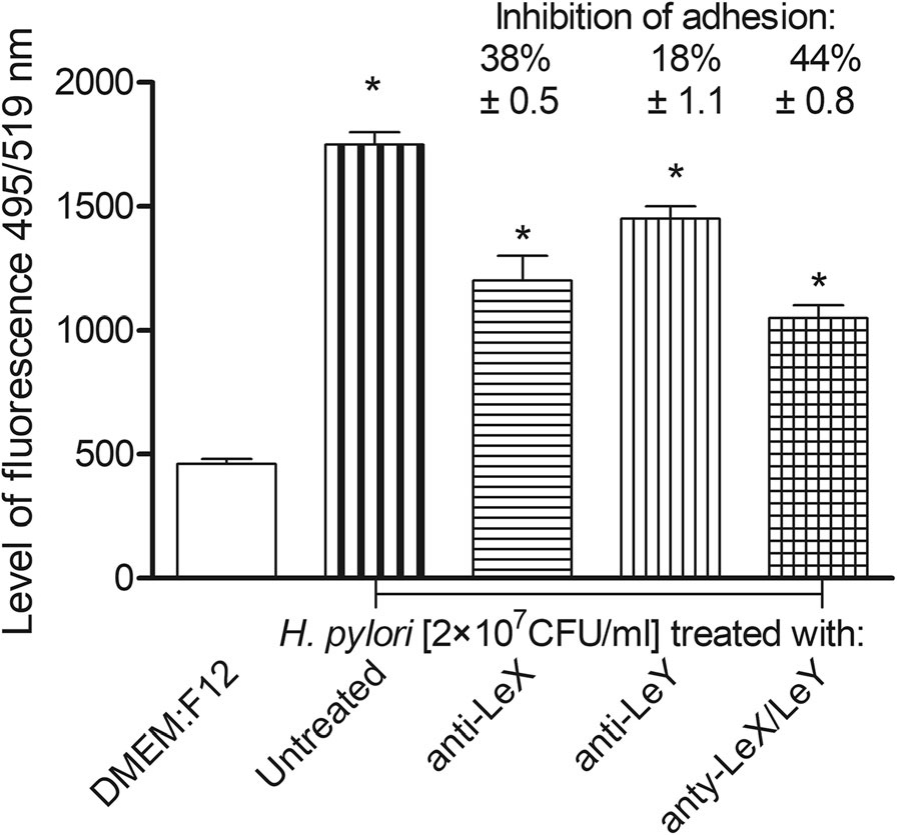
Involvement of *H. pylori* Lewis (Le) X or LeY determinents in adhesion to guinea pig primary gastric epithelial cells. Binding of *H. pylori* to gastric epithelial cells was evaluated by imaging *H. pylori* stained with anti-*H. pylori* antibodies (Ab) conjugated with fluoresceine isothiocyanate (FITC). Gastric epithelial cells were cocultured for 2 h with live *H. pylori* (2 × 10^7^ colony forming units –CFU/ml) non treated or treated for 30 min with anti-LeX, anti-LeY or both types of antibodies. The intensity of fluorescence was measured in a fluorescence reader at 495 nm excitation/519 nm emission, mean values ±SD. * Statistical significance for cells exposed to *H. pylori* untreated with anti-LeX or anti-LeX and anti-LeY antibodies, *vs* cells exposed to *H. pylori,* treated such antibodies, *p* < 0.05 in the non parametric *U* Mann-Whitney test

Because LPS of *H. pylori* strain used in this study contains LeX and LeY determinants we asked whether these determinants mediate bacterial binding to primary gastric epithelial cells. For binding experiments *H. pylori* untreated or treated with anti-LeX or anti-LeY antibodies were used. The binding effectiveness of *H. pylori* pre-treated with anti-LeX alone or in combination with anti-LeY antibodies was diminished significantly, 38 ± 0.5% and 44 ± 0.8%, respectively, *p* < 0.05 in U Mann-Whitney test (Fig. 3)

## Discussion

Since the description of *H. pylori* for the first time by R. Warren and B. Marshall in 1983, many aspects related to the host gastric epithelial cells colonization and pathogenesis of *H. pylori* have been intensively studied in order to understand the mechanisms of infection especially in view of its chronic nature and pathological consequences [7, 62–65]. Studies on the pathogenesis of *H. pylori* infections require appropriate models, both cellular in vitro models and in vivo animal models [66–68]. The most suitable model is *Caviae porcellus* (guinea pig), due to the anatomy and physiology of its stomach, which is similar to the human stomach, lack of natural *H. pylori* infection, ability to produce a homologue of human proinflammatory interleukin (IL) 8, and to develop both humoral and cellular specific immune responses to these bacteria as well as the need of external source of vitamin C [67, 68]. The phenomenon of *H. pylori* adhesion to the surface of gastric epithelial cells is crucial for colonization and maintenance of infection, which is accompanied by a chronic inflammatory reaction. The aim of this study was to evaluate the role of *H. pylori* and their solube components in MUC5AC production by gastric epithelial cells and the exposure of LeX/LeY determinants and their role in the attachment of these bacteria to the gastric tissue. Whether soluble components of these bacteria modulate the production of mucin components, including Lewis antigens and how they favor colonization of the gastric tissue and the maintenance of infection by these bacteria is not completely understood. In this study we used primary gastric epithelial cells derived from the guinea pig stomach as well as an in vivo model of experimental infection in these animals, lasting for 7 or 28 days, to follow the production of MUC5AC and deposition of LeX/LeY in the milieu of selected *H. pylori* components in cell cultures or in response to gastric tissue infection. The question was whether there is a relationship between the production of MUC5AC and LeX/LeY deposition, and the binding effectiveness of *H. pylori* to gastric epithelial cells. Mucin MUC5AC was suggested to be the main source of host receptors for *H. pylori* during colonization of gastric tissue in humans [69]. We showed that MUC5AC was produced by guinea pig primary gastric epithelial cells after 24 h incubation with *H. pylori* antigens used in this study such as: GE, CagA, UreA, LPS and after 2 h exposure to live bacteria. MUC5AC was most intensively produced in response to stimulation with *H. pylori* LPS but not *E. coli* LPS. Probably this is because *H. pylori* LPS contains fucosylated oligosaccharide antigens identical to human antigens Lea, Leb, sLeX, sLeY, H type 1 and A, B antigens of the ABO blood group system, whose expression changes during the inflammatory response [35, 70–73]. *Van den Brink* et al. (2000) and later* Park* et al. (2015) confirmed the expression of MUC5AC in the human stomach and demonstrated the importance of MUC5AC in gastric mucosa colonization by *H. pylori* [74]. In humans binding of *H. pylori* with Leb and sialylated determinants of MUC5AC such as sLeX in the gastric mucosa is mediated by surface *H. pylori* adhesins such as BabA and SabA, respectively [27, 28, 31, 36, 75]. *H. pylori* with a deletion of the *babA* gene was found clearly less effective in binding mucin than wild strain [42]. In our study the increased production of MUC5AC in in vitro model of guinea pig primary gastric epithelial cells was related to elevated deposition of Lewis determinants: LeX and LeY in response to live *H. pylori*, UreA and *H. pylori* LPS. Other *H. pylori* components such as GE or CagA and the reference LPS *E. coli* increased the deposition of LeX rather than LeY. Considering the islad-like character of *H. pylori* infection it is possible that different *H. pylori* soluble components may influence locally the production of gastric mucus containing MUC5AC as well as LeX/Y, which can be involved in binding these bacteria in dose dependent manner. Interaction of *H. pylori* with mucins and colonization effectiveness were also confirmed by the study on three cell lines grown on “transwel” type filters: HT29 (non-mucin secreting line), HT29-MTX (native, mucin secreting), HT29- MTX-E13 (containing an adherent mucus layer). *H. pylori* colonized the HT29-MTX-E13 cells most intensively, while the HT29 line was not colonized by these bacteria [76]. *Park* et al. (2015) showed *H. pylori* binding to mucins (including MUC5AC) isolated from gastric juice and gastric biopsies from patients with functional dyspepsia [74]. *Perrais* et al. (2014) using in vitro model of gastric cancer cells KATO III looked at the molecular mechanism driven by *H. pylori* that upregulate mucin gene expression in the stomach [77]. The strong MUC5AC gene expression in cells infected with UreB^−^ isogenic mutant but not with wild bacteria producing urease was showed. It indicated that *H. pylori* urease may downregulate MUC5AC expression in already transformed gastric cancer cells although this phenomenon does not have to refer to primary cells, which may possess diferent mechanisms.

In our experimental model of *H. pylori* infection in guinea pigs, the infection was confirmed, both 7 and 28 days after the last inoculation by histological, molecular (*ureC*, *cagA* PCR) and serological methods (ELISA for anti-*H. pylori* IgG and immunoenzymiatic test for the detection of *H. pylori* antigens in stool samples). Gastric mucosa of infected animals was infiltrated by eosinophils and lymphocytes to the higher level 28 than 7 days after inoculation indicating a development of chronic inflammatory response during the course of infection. In this model we focused on the MUC5AC production and LeX exposure during *H. pylori* infection, which was selected on the basis of in vitro experiments on the guinea pig primary gastric epithelial cells showing a domination of LeX rather than LeY deposition in response to live *H. pylori* or soluble bacterial compounds. These biomarkers were increased, however, more effectively after 7 than 28 days post inoculation. Stronger mucin production and LeX deposition in the first stage of infection probably is necessary for these bacteria for colonization of gastric niche, whereas during the chronic phase of infection interactions of *H. pylori* with gastric epithelial cells rather than with gastric mucin are more important to the maintenance of infection. Downregulation of mucin production, which reperesent the first line of host defence against infectious agents in the later phase of infection can protect bacteria from antimicrobial propertis of mucin. *Park *et al. (2015) showed that in patients with chronic *H. pylori* infection the MUC5AC production was even lower than in uninfected individuals [74]. In this study using guinea pig primary gastric epithelial cells and tissue sections of infected animals, we showed that increased MUC5AC production and Lewis antigens deposition, especially of LeX, in the milieu of *H. pylori* and their soluble antigens or during the experimental infection significantly improved the colonization process. Recently *Naughton* et al. 2013, showed an intensive binding of three *H. pylori* strains to mucins in the rat gastric and duodenal mucosa and duodenum [31]. *Navabi *et al. (2013) investigated the production rate and turnover of newly synthesised mucin in mice and analyzed the effects of early colonization and chronic infection with *H. pylori* [78]. They evaluated metabolic incorporation of an azido GalNac analog (GalNaz) in the experiments with the whole animals infected with *H. pylori* strain SS1 during early colonization (7 days) and chronic infection (90 days). The *H. pylori* infection in mice reduced the rate of MUC1 but not MUC5AC. In our guinea pig model the increased MUC5AC production during early phase of infection was linked with enhanced deposition of LeX in gastric tissue whereas diminished mucin and LeX production was showed during chronic phase of infection. This indicate that *H. pylori* can modulate the environment of the stomach by impairement of the defence mechanisms, including mucin production. *Byrd* et al. (2000) showed on KATO III cells that MUC5AC production in response to *H. pylori* was not reversible within 24 h [79]. In early phase of infection *H. pylori* need mucin receptors for tight adhesios. By contrast, downregulation of mucin production during later stage of *H. pylori*-host interaction may diminish clearence of pathogens and promote the maintenance of infection. The results of this study using antibodies that block the availability of MUC5CA and LeX or LeY for *H. pylori* have indicated the importance of these components of gastric mucosa in the adhesion of these bacteria to *Caviae porcellus* gastric epithelial cells. In conclusion, the experiments carried out in vitro on a cellular model and in vivo on guinea pigs infected with *H. pylori* confirmed the role of MUC5AC containing LeX and LeY determinants in *H. pylori* binding to gastric epithelial cells. It was also showed that both live *H. pylori* and soluble components of these bacteria were able to stimulate the production by gastric epithelial cells of MUC5AC containing LeX and LeY determinants and promote colonization. The elevation of MUC5AC production and modulation of deposition of LeX or LeY in gastric tissue in response to *H. pylori* antigens can be an important mechanism for the maintenance of infection, induction of chronic inflammatory response and deleterious effects on the level of gastric barrier during *H. pylori* infection. It is worth of mentioning that Lewis components, which are present in LPS of *H. pylori*, can mediate the interaction of these bacteria with gastric epithelial cells. It was confirmed by impairement of adhesion to guinea pig gastric epithelial cells of *H. pylori* treated with anti-LeX, anti-LeY or both types of antibodies. Previously, it was showed that LeX and LeY of *H. pylori* were involved in phagocytosis of these bacteria and induction of the immune complexes LewisX/Y-anti-LewisX/Y IgGwith proinflammatory potential [80]. In conclusion LeX/LeY determinants present in gastric mucin as well as *H. pylori* LeX/LeY determinants can be used by these bacteria for colonization of gastric mucosa of the host. *Skoog* et al., (2012) showed that expression of genes encoding important virulence factors of *H. pylori* such as BabA, SabA, CagA, UreA and flagellin is co-regulated in response to host mucins binding [81]. For instance the expression of the BabA and SabA adhesins, which is tightly regulated on this way, potentially permits the bacteria to adapt to the host gastric mucosa glycosylation during infection and to evoid the negative effects of inflammatory response [82].

## Conclusions

MUC5AC production and deposition of LeX/LeY determinants were significantly elevated in response to live *H. pylori* or soluble components of these bacteria and positively correlated with *H. pylori* adhesion to the gastric mucosa on experimental model of guinea pig gastric epithelial cells in vitro and in vivo. Upregulation of MUC5AC/LeX/LeY by *H. pylori* in early phase of infection can promote successful colonization of gastric niche whereas downregulation of MUC5AC/LeX/LeY persistent infection and development of chronic inflammatory response, which can be followed by deleterious effects locally in the gastric tissue and potentially also systemically.

## Abbreviations

AlpA: adherence-associated lipoprotein A
AlpB: adherence-associated lipoprotein B
BabA: blood antigen-binding adhesin A
BSA: bovine serum albumin
CagA: cytotoxin associated gene A antigen
CFU: colony forming unit
cRPMI: complete RPMI-1640 culture medium
DAPI: 4′,6-diamidino-2-phenylindole
DMEM: Dulbecco’s Modified Eagle’s medium
DMSO: dimethyl sulfoxide
ECM: extracellular matrix
EDTA: ethylenediaminetetraacteic acid
ELISA: Enzyme-likned immunosorbent assay
FCS: fetal calf serum
FITC: fluorescein isothiocyanate
GE: glycine acid extract
H&E: hematoxilin and eosin staining
HBSS: Hank’s Balanced Salt Solution
HEPES: N-2-hydroxyethylpiperazine-N-2-ethane sulfonicacid
HLO: *Helicobacter-*like organisms
HopZ: *Helicobacter* outer membrane protein Z
Le: Lewis antigen
LPS: lipopolysaccharide
MALT: mucosa-associated lymphoid tissue
MUC5AC: mucin 5
OpiA: outer membrane protein A
PBS: phosphate buffered saline
PCR: polymerase chain reaction
SabA: sialic acid binding adhesin
SD: standard deviation
TBS: Trypticase Soy Broth
TBST: Tris-Buffered Saline with Tween 20
VacA: vacuolating cytotoxin A
